# Aneurism of the Mesenteric Artery

**Published:** 1887-07

**Authors:** Richard W. Burke

**Affiliations:** Station Chief Veterinary Hospital, Cawnpore, India


### Art. XXII.—ANEURISM OF THE MESENTERIC ARTERY.

BY RICHARD W. BURKE, V.S., A.V.D.

*Station Chief Veterinary Hospital, Cawnpore, India.*

*History.*—Rigot in 1829, and Hering in 1830, were the first to observe that colic in the horse was in many cases due to worm-aneurism; and Schutt a few years later corroborated their observations. Bruckmüller carefully examined this subject in a number of horses, and found that very few of them are ever free from it. He confirmed the observations made by other veterinarians, that a great relationship between aneurism of the mesenteric artery, and colic cannot be denied. M. Bouley (*Dictionnaire Pratique*, 1856, *p.* 548), showed that hæmorrhagic and intermittent colic are dependent on aneurism of the mesenteric artery. Hahn, in 1862, considered periodic colic was due to aneurism of the mesenteric artery. Cornevin, in 1869, described a very interesting case of thrombatic colic with stoppage of the iliac and cæcal arteries. Since these observations, the condition has become familiar to all practitioners.

In oft-recurring cases of colic in the horse, aneurism of the mesenteric artery should be suspected. It frequently also escapes detection until post mortem, as in many horses aneurism of the mesenteric artery is seldom absent, even though there were no symptoms of its presence during life; this particularly so in the ass. General experience has now established this fact beyond a doubt. The works of Bruckmüller, Röll, Friedberger, Fröhner, Bollinger and others, show that in Germany 90 to 94 per cent. of the horses have aneurism of the mesenteric artery. Ellenberger noted that in eighty-five dissections he made, there was only one that was free from aneurism of the mesenteric artery. This aneurism is seen in young foals at birth, as well as in old horses, and especially in the anterior mesenteric artery.

*Causation.*—The presence of the worms, — variety *Strongylus armatus* of Rudulphi—is the cause of the chronic changes in the walls of the mesenteric artery, through which a thrombus results. Through lesions in the intima, a chronic traumatic endo-arteritis is set up, producing aneurism.

*Patho-Anatomy.*—The walls of the aneurism are thickened, (especially the media with the enveloping mesentery) long-oval and flattish, or spindle-shaped; less frequently it is sacculated, and of the size of a man's head. The intima shows the greatest variety of inflammation and degenerative changes, such as swellings, fatty and calcareous degenerations, becoming into knots and even sloughs resulting, with subsequent fibroid contraction. The contents are made up of a laminated thrombus with canals or perforations through the latter. The lumen of the aneurism is obstructed, and the thrombus may travel into the branching arteries, and sometimes even into the aorta. The clot is usually hard, knotty and brittle, with purulent softening in its interior. Besides the presence of the thrombus, a worm larva of the strongylus armatus will be found, generally nine in each aneurism (Bollinger, *Wochenschrift fur Thierheilkunde und Viehzucht*, 1873, *p.* 22), which cause the chronic changes of the intima, through which a thrombus results. On account of the stoppage of blood-flow for the nutrition of the intestinal walls, a threefold danger is in operation to produce colic : first, the lumen of the thrombus becoming completely blocked up may lead to stoppage of blood-flow to all the arteries beyond it ; or, secondly, the thrombus may migrate to one of the branch arteries ; or, lastly, detachments of the thrombus may be carried along as emboli, with the blood, to the other branches of the intestinal arteries. In all these cases the thrombus fixes itself and prevents circulation in all collateral branches ; then in the blocked up capillaries anæmia results ; and from the rebound of the blood current from the point of obstruction serous and bloody transudation into the intestinal walls, and even into the lumen of the intestines, results (the so-called "sero-hæmorrhagic infarct"), which leads to paralysis of the intestine. Hence stagnation in the intestinal canal results, which, undergoing decomposition, creates gas formation and colic. The paralysis leads to inflammation and rupture of the intestine in many cases, and death.

Professor Lustig (*Deut. Zeitschrift fur Thiermed u. vergl. Pathologie, Bd. i, Heft ii and iii*, 1879), of the Hanover Verterinary School, describes an interesting case of embolic nephritis with worm-aneurism in a horse, which on post mortem examination showed thrombi containing strongyli in the renal artery, and worm-aneurism of the anterior mesenteric artery, the colic artery, the ilio-cœcal artery, with thrombus in the pulmonary artery and hypertrophy of the heart.

